# 1,4a,7-Trimethyl-7-vinyl-1,2,3,4,4a,4b,5,6,7,8,10,10a-dodeca­hydro­phenanthrene-1-carboxylic acid

**DOI:** 10.1107/S1600536809019308

**Published:** 2009-05-29

**Authors:** Zhen-Dong Zhao, Yu-Xiang Chen, Yu-Min Wang, Liang-Wu Bi

**Affiliations:** a Institute of Chemical Industry of Forest Products, Chinese Academy of Forestry, Nanjing 210042, People’s Republic of China

## Abstract

The title compound, also known as isopimaric acid, C_20_H_30_O_2_, was isolated from slash pine rosin. There are two unique mol­ecules in the unit cell. The two cyclo­hexane rings have classical chair conformations. The cyclo­hexene ring represents a semi-chair. The mol­ecular conformation is stabilized by weak intra­molecular C—H⋯O hydrogen-bonding inter­actions. The mol­ecules are dimerized through their carboxyl groups by O—H⋯O hydrogen bonds, forming *R*_2_^2^(8) rings.

## Related literature

For physical and spectroscopic analysis, see: Baldwin *et al.* (1958 ▶); Harris & Sanderson (1948 ▶). For biological activities, see: Smith *et al.* (2005 ▶); Russo *et al.* (2007 ▶).
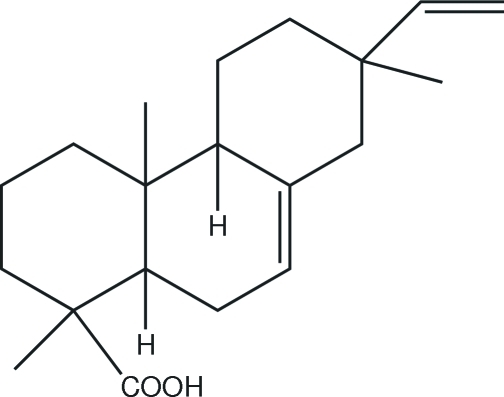


## Experimental

### 

#### Crystal data

C_20_H_30_O_2_
*M* *_r_* = 302.44
Orthorhombic, 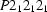

*a* = 11.624 (2) Å
*b* = 11.803 (2) Å
*c* = 25.698 (5) Å
*V* = 3525.7 (12) Å^3^
*Z* = 8
Mo *K*α radiation
μ = 0.07 mm^−1^
*T* = 293 K
0.20 × 0.10 × 0.10 mm

#### Data collection

Enraf–Nonius CAD-4 diffractometer
Absorption correction: ψ scan (North *et al.*, 1968 ▶) *T*_min_ = 0.986, *T*_max_ = 0.993
7031 measured reflections
6382 independent reflections
2613 reflections with *I* > 2σ(*I*)
*R*_int_ = 0.088
3 standard reflections every 200 reflections intensity decay: 1%

#### Refinement

*R*[*F* ^2^ > 2σ(*F* ^2^)] = 0.069
*wR*(*F* ^2^) = 0.150
*S* = 1.00
3598 reflections
385 parameters
1 restraint
H-atom parameters constrained
Δρ_max_ = 0.24 e Å^−3^
Δρ_min_ = −0.26 e Å^−3^

### 

Data collection: *CAD-4 Software* (Enraf–Nonius, 1989 ▶); cell refinement: *CAD-4 Software*; data reduction: *XCAD4* (Harms & Wocadlo, 1995 ▶); program(s) used to solve structure: *SHELXS97* (Sheldrick, 2008 ▶); program(s) used to refine structure: *SHELXL97* (Sheldrick, 2008 ▶); molecular graphics: *SHELXTL* (Sheldrick, 2008 ▶); software used to prepare material for publication: *SHELXTL*.

## Supplementary Material

Crystal structure: contains datablocks I, global. DOI: 10.1107/S1600536809019308/at2789sup1.cif

Structure factors: contains datablocks I. DOI: 10.1107/S1600536809019308/at2789Isup2.hkl

Additional supplementary materials:  crystallographic information; 3D view; checkCIF report

## Figures and Tables

**Table 1:** Hydrogen-bond geometry (Å, °)

*D*—H⋯*A*	*D*—H	H⋯*A*	*D*⋯*A*	*D*—H⋯*A*
O2—H2*B*⋯O4^i^	0.82	1.84	2.653 (7)	168
O3—H3*D*⋯O1^ii^	0.82	1.85	2.655 (7)	168
C11—H11*A*⋯O1	0.98	2.34	2.764 (7)	105
C32—H32*B*⋯O4	0.97	2.49	3.081 (9)	119

Symmetry codes: (i) 
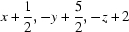
; (ii) 
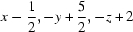
.

**Fig. 1.**
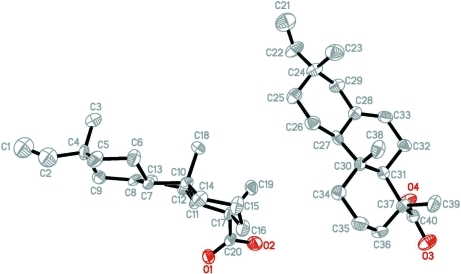
A view of the molecular structure of (I), showing displacement ellipsoids at the 30% probability level.

**Fig. 2.**
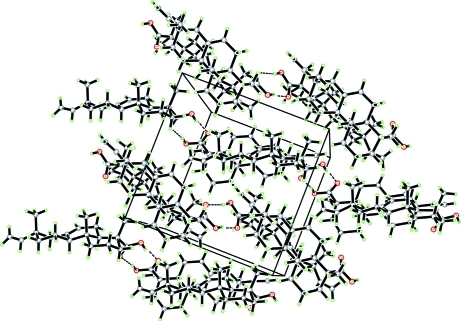
A view of the packing of the molecules dimerized through their carboxyl groups by O—H···O hydrogen bonds, forming R22(8) rings.

## supplementary crystallographic information

### Comment

The title compound has been isolated from slash pine rosin. It was identified as isopimaric acid on the basis of the comparison of its physical and spectral data with literature values (Baldwin *et al.*, 1958; Harris *et al.*, 1948). Isopimaric acid exhibits a wide range of biological activities such as antibacterial activity (Smith *et al.*, 2005), BK channels activity (Russo *et al.*, 2007). Although much attention has been paid to the bioactivities of isopimaric acid, the crystal structure of the title compound has not yet been reported. In this work, we describe the crystal structure of the title compound (I).

The molecular structure of (I) is shown in Fig. 1 and its crystal packing in Fig.2. The atoms of C11, C12 in the cyclohexene ring and the atoms C10, C14 in the conjoint cyclohexane ring are in the same plane. The atoms of C11, C12 and the atoms of C16, C17 in the cyclohexane ring are in another plane. The dihedral angle of the two planes is 134.4 degree. The three methyl groups attached to the cyclohexane rings are in axial positions and in the same direction. The molecular conformation is stabilized by C—H···O intra-molecular hydrogen bonding interactions (Table 1). The molecules are dimerized through their carboxyl groups by O—H···O hydrogen bonds, forming *R*_2_^2^(8) rings (Fig. 2).

### Experimental

A mixture of slash pine rosin (150 g), acetone (300 ml) and 2-amino-2-methyl-1-propanol (0.1 mol) was stirred at room temperature for 2 h and then filtrated. The residue was recrystallized with 95% ethanol. The crystal obtained from the solution was acidified by 5% hydrochloric acid solution and then dissolved in ether. The solution was washed with water until it was neutral, dryed with sodium sulfate and then concentrated. The residue was recrystallized with acetone and the title compound was obtained as colourless solid.

### Refinement

All H atoms were placed geometrically with C—H = 0.93–0.98 Å and O—H = 0.82 Å, and included in the refinement in riding motion approximation with *U*_iso_(H) = 1.2 or 1.5*U*_eq_ of the carrier atom. Because of no atom heavier than Si present in the structure, in the absence of significant anomalous dispersion effects, so Friedel pairs were averaged.

### Crystal data

**Table:**

C_20_H_30_O_2_	*D*_x_ = 1.140 Mg m^−^^3^
*M**_r_* = 302.44	Melting point: 436 K
Orthorhombic, *P*2_1_2_1_2_1_	Mo *K*α radiation, λ = 0.71073 Å
Hall symbol: P 2ac 2ab	Cell parameters from 25 reflections
*a* = 11.624 (2) Å	θ = 9–12°
*b* = 11.803 (2) Å	µ = 0.07 mm^−^^1^
*c* = 25.698 (5) Å	*T* = 293 K
*V* = 3525.7 (12) Å^3^	Quadrate, colourless
*Z* = 8	0.20 × 0.10 × 0.10 mm
*F*(000) = 1328	

### Data collection

**Table:**

Enraf–Nonius CAD-4 diffractometer	2613 reflections with *I* > 2σ(*I*)
Radiation source: fine-focus sealed tube	*R*_int_ = 0.088
graphite	θ_max_ = 25.3°, θ_min_ = 1.6°
ω/2θ scans	*h* = 0→13
Absorption correction: ψ scan (North *et al.*, 1968)	*k* = 0→14
*T*_min_ = 0.986, *T*_max_ = 0.993	*l* = −30→30
7031 measured reflections	3 standard reflections every 200 reflections
6382 independent reflections	intensity decay: 1%

### Refinement

**Table:**

Refinement on *F*^2^	Primary atom site location: structure-invariant direct methods
Least-squares matrix: full	Secondary atom site location: difference Fourier map
*R*[*F*^2^ > 2σ(*F*^2^)] = 0.069	Hydrogen site location: inferred from neighbouring sites
*wR*(*F*^2^) = 0.150	H-atom parameters constrained
*S* = 1.00	*w* = 1/[σ^2^(*F*_o_^2^) + (0.045*P*)^2^] where *P* = (*F*_o_^2^ + 2*F*_c_^2^)/3
3598 reflections	(Δ/σ)_max_ < 0.001
385 parameters	Δρ_max_ = 0.24 e Å^−^^3^
1 restraint	Δρ_min_ = −0.26 e Å^−^^3^

### Special details

**Table:**

Geometry. All e.s.d.'s (except the e.s.d. in the dihedral angle between two l.s. planes) are estimated using the full covariance matrix. The cell e.s.d.'s are taken into account individually in the estimation of e.s.d.'s in distances, angles and torsion angles; correlations between e.s.d.'s in cell parameters are only used when they are defined by crystal symmetry. An approximate (isotropic) treatment of cell e.s.d.'s is used for estimating e.s.d.'s involving l.s. planes.
Refinement. Refinement of *F*^2^ against ALL reflections. The weighted *R*-factor *wR* and goodness of fit *S* are based on *F*^2^, conventional *R*-factors *R* are based on *F*, with *F* set to zero for negative *F*^2^. The threshold expression of *F*^2^ > σ(*F*^2^) is used only for calculating *R*-factors(gt) *etc*. and is not relevant to the choice of reflections for refinement. *R*-factors based on *F*^2^ are statistically about twice as large as those based on *F*, and *R*- factors based on ALL data will be even larger.

### Fractional atomic coordinates and isotropic or equivalent isotropic displacement parameters (Å^2^)

**Table:**

	*x*	*y*	*z*	*U*_iso_*/*U*_eq_	
O1	0.7921 (4)	0.8907 (4)	0.96412 (19)	0.0796 (17)	
O2	0.6441 (4)	1.0009 (4)	0.97857 (19)	0.0816 (16)	
H2B	0.6765	1.0386	0.9561	0.122*	
C1	0.9481 (9)	0.1236 (8)	1.0193 (3)	0.127	
H1A	0.8843	0.0857	1.0060	0.152*	
H1B	1.0204	0.0899	1.0178	0.152*	
C2	0.9370 (8)	0.2172 (8)	1.0385 (3)	0.110	
H2A	1.0080	0.2437	1.0499	0.132*	
C3	0.7282 (7)	0.2534 (6)	1.0540 (3)	0.088 (3)	
H3A	0.7268	0.1988	1.0817	0.132*	
H3B	0.7082	0.2170	1.0218	0.132*	
H3C	0.6738	0.3126	1.0612	0.132*	
C4	0.8463 (7)	0.3031 (6)	1.0497 (3)	0.064 (2)	
C5	0.8871 (7)	0.3611 (6)	1.0991 (3)	0.076 (2)	
H5A	0.9652	0.3881	1.0942	0.091*	
H5B	0.8874	0.3070	1.1275	0.091*	
C6	0.8086 (6)	0.4615 (5)	1.1131 (3)	0.069 (2)	
H6A	0.7309	0.4342	1.1187	0.083*	
H6B	0.8352	0.4957	1.1452	0.083*	
C7	0.8077 (5)	0.5512 (5)	1.0699 (2)	0.0449 (16)	
H7A	0.8864	0.5808	1.0676	0.054*	
C8	0.7810 (6)	0.4997 (5)	1.0180 (3)	0.0496 (17)	
C9	0.8475 (6)	0.3926 (5)	1.0056 (3)	0.066 (2)	
H9A	0.9267	0.4127	0.9981	0.080*	
H9B	0.8154	0.3588	0.9744	0.080*	
C10	0.7276 (6)	0.6559 (5)	1.0837 (2)	0.0461 (16)	
C11	0.7174 (6)	0.7258 (5)	1.0326 (2)	0.0469 (16)	
H11A	0.7965	0.7339	1.0199	0.056*	
C12	0.6560 (6)	0.6608 (5)	0.9911 (2)	0.0579 (19)	
H12A	0.6586	0.7027	0.9586	0.070*	
H12B	0.5759	0.6510	1.0007	0.070*	
C13	0.7126 (6)	0.5439 (5)	0.9835 (3)	0.0599 (19)	
H13A	0.6972	0.5035	0.9533	0.072*	
C14	0.7876 (6)	0.7254 (5)	1.1253 (2)	0.0577 (18)	
H14A	0.8674	0.7364	1.1154	0.069*	
H14B	0.7864	0.6839	1.1579	0.069*	
C15	0.7310 (7)	0.8406 (5)	1.1333 (3)	0.071 (2)	
H15A	0.7709	0.8811	1.1607	0.086*	
H15B	0.6518	0.8298	1.1441	0.086*	
C16	0.7336 (7)	0.9111 (6)	1.0836 (3)	0.071 (2)	
H16A	0.8129	0.9255	1.0739	0.085*	
H16B	0.6968	0.9835	1.0900	0.085*	
C17	0.6704 (6)	0.8497 (5)	1.0371 (2)	0.0523 (18)	
C18	0.6102 (5)	0.6120 (6)	1.1030 (3)	0.0601 (19)	
H18A	0.5611	0.6751	1.1109	0.090*	
H18B	0.6211	0.5670	1.1337	0.090*	
H18C	0.5752	0.5665	1.0764	0.090*	
C19	0.5398 (6)	0.8563 (6)	1.0466 (3)	0.071 (2)	
H19A	0.5171	0.9341	1.0500	0.106*	
H19B	0.5210	0.8162	1.0780	0.106*	
H19C	0.4999	0.8225	1.0178	0.106*	
C20	0.7059 (7)	0.9151 (5)	0.9899 (3)	0.0554 (19)	
O3	0.3506 (4)	1.4855 (4)	1.11796 (19)	0.0782 (16)	
H3D	0.3267	1.5163	1.0915	0.117*	
O4	0.2231 (4)	1.3579 (4)	1.09334 (19)	0.0715 (15)	
C21	0.0730 (7)	0.5800 (8)	1.1914 (4)	0.112 (3)	
H21A	0.0778	0.5751	1.2275	0.135*	
H21B	0.0414	0.5207	1.1724	0.135*	
C22	0.1091 (7)	0.6666 (6)	1.1685 (4)	0.087 (3)	
H22A	0.1017	0.6661	1.1325	0.104*	
C23	0.1430 (6)	0.7841 (7)	1.2495 (3)	0.090 (3)	
H23A	0.0621	0.7856	1.2569	0.134*	
H23B	0.1774	0.8540	1.2606	0.134*	
H23C	0.1781	0.7222	1.2678	0.134*	
C24	0.1616 (7)	0.7689 (6)	1.1909 (3)	0.064 (2)	
C25	0.2897 (6)	0.7684 (5)	1.1787 (3)	0.067 (2)	
H25A	0.3248	0.7052	1.1968	0.081*	
H25B	0.2993	0.7553	1.1417	0.081*	
C26	0.3541 (6)	0.8752 (5)	1.1930 (3)	0.065 (2)	
H26A	0.4335	0.8676	1.1819	0.078*	
H26B	0.3539	0.8831	1.2306	0.078*	
C27	0.3037 (5)	0.9826 (5)	1.1690 (2)	0.0489 (17)	
H27A	0.3218	0.9779	1.1318	0.059*	
C28	0.1740 (6)	0.9838 (6)	1.1720 (2)	0.0519 (18)	
C29	0.1117 (6)	0.8732 (5)	1.1632 (3)	0.066 (2)	
H29A	0.1107	0.8581	1.1261	0.080*	
H29B	0.0325	0.8825	1.1743	0.080*	
C30	0.3612 (5)	1.0954 (5)	1.1883 (3)	0.0486 (17)	
C31	0.2935 (5)	1.1915 (5)	1.1639 (2)	0.0467 (17)	
H31A	0.2870	1.1711	1.1270	0.056*	
C32	0.1699 (6)	1.1945 (6)	1.1837 (3)	0.0574 (19)	
H32A	0.1690	1.2197	1.2196	0.069*	
H32B	0.1255	1.2480	1.1633	0.069*	
C33	0.1167 (6)	1.0799 (6)	1.1800 (3)	0.067 (2)	
H33A	0.0372	1.0752	1.1834	0.081*	
C34	0.4849 (5)	1.0947 (6)	1.1698 (3)	0.061 (2)	
H34A	0.4870	1.0785	1.1328	0.074*	
H34B	0.5266	1.0351	1.1877	0.074*	
C35	0.5446 (6)	1.2105 (6)	1.1803 (3)	0.074 (2)	
H35A	0.5418	1.2283	1.2171	0.089*	
H35B	0.6246	1.2073	1.1696	0.089*	
C36	0.4813 (5)	1.3000 (6)	1.1494 (3)	0.061 (2)	
H36A	0.4862	1.2810	1.1127	0.073*	
H36B	0.5199	1.3721	1.1543	0.073*	
C37	0.3535 (6)	1.3142 (5)	1.1640 (2)	0.0509 (17)	
C38	0.3546 (6)	1.0973 (5)	1.2479 (2)	0.067 (2)	
H38A	0.3887	1.1660	1.2607	0.101*	
H38B	0.3954	1.0334	1.2618	0.101*	
H38C	0.2755	1.0938	1.2586	0.101*	
C39	0.3395 (7)	1.3764 (5)	1.2144 (3)	0.075 (2)	
H39A	0.3785	1.4480	1.2124	0.112*	
H39B	0.3719	1.3322	1.2422	0.112*	
H39C	0.2592	1.3889	1.2210	0.112*	
C40	0.3037 (6)	1.3882 (6)	1.1227 (3)	0.0529 (18)	

### Atomic displacement parameters (Å^2^)

**Table:**

	*U*^11^	*U*^22^	*U*^33^	*U*^12^	*U*^13^	*U*^23^
O1	0.085 (4)	0.058 (3)	0.096 (4)	0.016 (3)	0.042 (3)	0.013 (3)
O2	0.082 (4)	0.073 (3)	0.089 (4)	0.007 (3)	0.022 (3)	0.030 (3)
C1	0.137	0.107	0.137	0.000	0.000	0.000
C2	0.110	0.110	0.110	0.000	0.000	0.000
C3	0.093 (7)	0.057 (5)	0.114 (7)	−0.008 (5)	0.022 (6)	0.012 (5)
C4	0.079 (6)	0.043 (4)	0.070 (5)	0.026 (4)	0.023 (5)	0.006 (4)
C5	0.094 (6)	0.058 (5)	0.076 (5)	0.002 (5)	−0.002 (5)	0.019 (5)
C6	0.087 (6)	0.056 (4)	0.064 (5)	0.017 (4)	0.005 (4)	0.005 (4)
C7	0.049 (4)	0.035 (3)	0.051 (4)	0.001 (3)	0.000 (3)	0.000 (3)
C8	0.063 (4)	0.031 (3)	0.054 (4)	−0.004 (4)	−0.001 (4)	0.014 (3)
C9	0.060 (5)	0.057 (4)	0.082 (5)	−0.007 (4)	0.012 (4)	−0.015 (4)
C10	0.052 (4)	0.041 (3)	0.046 (4)	−0.002 (4)	−0.003 (3)	0.005 (3)
C11	0.057 (4)	0.036 (3)	0.048 (4)	−0.004 (3)	−0.004 (4)	0.004 (3)
C12	0.073 (5)	0.049 (4)	0.052 (4)	0.004 (4)	−0.005 (4)	0.015 (3)
C13	0.073 (5)	0.047 (4)	0.059 (5)	−0.012 (4)	−0.004 (4)	−0.005 (4)
C14	0.070 (5)	0.059 (4)	0.044 (4)	0.000 (4)	−0.009 (4)	0.005 (4)
C15	0.101 (6)	0.055 (4)	0.058 (5)	0.020 (5)	0.000 (4)	−0.004 (4)
C16	0.088 (6)	0.056 (4)	0.068 (5)	0.010 (5)	0.005 (4)	−0.002 (4)
C17	0.072 (5)	0.039 (4)	0.045 (4)	0.003 (4)	0.008 (4)	−0.003 (3)
C18	0.052 (4)	0.062 (5)	0.066 (5)	−0.001 (4)	0.019 (4)	0.000 (4)
C19	0.072 (5)	0.062 (5)	0.078 (5)	0.025 (4)	0.016 (4)	0.022 (4)
C20	0.070 (5)	0.033 (4)	0.064 (5)	0.008 (4)	−0.002 (4)	0.018 (4)
O3	0.097 (4)	0.057 (3)	0.080 (4)	−0.014 (3)	−0.028 (3)	0.019 (3)
O4	0.075 (3)	0.057 (3)	0.083 (3)	−0.011 (3)	−0.036 (3)	0.025 (3)
C21	0.121 (8)	0.099 (7)	0.116 (8)	−0.031 (7)	−0.020 (7)	−0.016 (7)
C22	0.092 (7)	0.055 (5)	0.113 (8)	−0.025 (5)	−0.032 (6)	0.006 (5)
C23	0.096 (6)	0.091 (6)	0.082 (6)	−0.002 (6)	0.001 (5)	0.023 (5)
C24	0.075 (6)	0.052 (5)	0.065 (5)	−0.016 (4)	−0.012 (4)	0.010 (4)
C25	0.075 (6)	0.055 (5)	0.072 (5)	−0.005 (4)	−0.017 (5)	−0.004 (4)
C26	0.051 (4)	0.065 (5)	0.079 (5)	−0.003 (4)	−0.004 (4)	0.007 (4)
C27	0.045 (4)	0.062 (4)	0.039 (4)	0.022 (4)	0.003 (3)	0.009 (3)
C28	0.061 (5)	0.050 (4)	0.044 (4)	0.002 (4)	−0.008 (3)	0.007 (4)
C29	0.073 (5)	0.060 (5)	0.066 (5)	−0.013 (4)	−0.007 (4)	0.004 (4)
C30	0.045 (4)	0.045 (4)	0.056 (4)	0.002 (3)	0.000 (4)	−0.005 (3)
C31	0.043 (4)	0.053 (4)	0.044 (4)	0.001 (3)	−0.003 (3)	−0.004 (3)
C32	0.058 (5)	0.057 (5)	0.057 (4)	0.003 (4)	0.012 (4)	0.014 (4)
C33	0.055 (5)	0.065 (5)	0.082 (6)	0.000 (4)	0.016 (4)	0.023 (5)
C34	0.060 (5)	0.062 (5)	0.062 (5)	−0.002 (4)	−0.005 (4)	−0.003 (4)
C35	0.056 (5)	0.073 (5)	0.092 (6)	−0.006 (4)	0.007 (5)	0.011 (5)
C36	0.053 (5)	0.052 (4)	0.076 (5)	−0.015 (4)	−0.010 (4)	0.014 (4)
C37	0.060 (4)	0.045 (4)	0.048 (4)	−0.004 (4)	−0.009 (4)	−0.001 (3)
C38	0.103 (6)	0.055 (4)	0.043 (4)	0.011 (5)	−0.008 (4)	−0.004 (4)
C39	0.104 (6)	0.051 (4)	0.070 (5)	0.001 (5)	0.000 (5)	−0.011 (4)
C40	0.050 (4)	0.052 (5)	0.057 (4)	0.003 (4)	−0.002 (4)	0.003 (4)

### Geometric parameters (Å, °)

**Table:**

O1—C20	1.235 (7)	O3—C40	1.278 (7)
O2—C20	1.276 (7)	O3—H3D	0.8200
O2—H2B	0.8200	O4—C40	1.256 (7)
C1—C2	1.217 (8)	C21—C22	1.252 (10)
C1—H1A	0.9300	C21—H21A	0.9300
C1—H1B	0.9300	C21—H21B	0.9300
C2—C4	1.491 (10)	C22—C24	1.470 (9)
C2—H2A	0.9300	C22—H22A	0.9300
C3—C4	1.496 (9)	C23—C24	1.532 (10)
C3—H3A	0.9600	C23—H23A	0.9600
C3—H3B	0.9600	C23—H23B	0.9600
C3—H3C	0.9600	C23—H23C	0.9600
C4—C5	1.518 (10)	C24—C25	1.522 (10)
C4—C9	1.549 (9)	C24—C29	1.536 (9)
C5—C6	1.538 (9)	C25—C26	1.511 (8)
C5—H5A	0.9700	C25—H25A	0.9700
C5—H5B	0.9700	C25—H25B	0.9700
C6—C7	1.534 (8)	C26—C27	1.528 (8)
C6—H6A	0.9700	C26—H26A	0.9700
C6—H6B	0.9700	C26—H26B	0.9700
C7—C8	1.498 (8)	C27—C28	1.510 (8)
C7—C10	1.587 (8)	C27—C30	1.570 (8)
C7—H7A	0.9800	C27—H27A	0.9800
C8—C13	1.299 (8)	C28—C33	1.331 (8)
C8—C9	1.516 (8)	C28—C29	1.510 (8)
C9—H9A	0.9700	C29—H29A	0.9700
C9—H9B	0.9700	C29—H29B	0.9700
C10—C14	1.517 (8)	C30—C34	1.514 (8)
C10—C18	1.542 (8)	C30—C31	1.516 (8)
C10—C11	1.555 (7)	C30—C38	1.536 (8)
C11—C12	1.496 (8)	C31—C32	1.525 (8)
C11—C17	1.565 (8)	C31—C37	1.607 (8)
C11—H11A	0.9800	C31—H31A	0.9800
C12—C13	1.541 (8)	C32—C33	1.491 (9)
C12—H12A	0.9700	C32—H32A	0.9700
C12—H12B	0.9700	C32—H32B	0.9700
C13—H13A	0.9300	C33—H33A	0.9300
C14—C15	1.524 (8)	C34—C35	1.556 (8)
C14—H14A	0.9700	C34—H34A	0.9700
C14—H14B	0.9700	C34—H34B	0.9700
C15—C16	1.525 (9)	C35—C36	1.514 (9)
C15—H15A	0.9700	C35—H35A	0.9700
C15—H15B	0.9700	C35—H35B	0.9700
C16—C17	1.578 (9)	C36—C37	1.541 (9)
C16—H16A	0.9700	C36—H36A	0.9700
C16—H16B	0.9700	C36—H36B	0.9700
C17—C20	1.496 (9)	C37—C40	1.491 (8)
C17—C19	1.540 (8)	C37—C39	1.498 (8)
C18—H18A	0.9600	C38—H38A	0.9600
C18—H18B	0.9600	C38—H38B	0.9600
C18—H18C	0.9600	C38—H38C	0.9600
C19—H19A	0.9600	C39—H39A	0.9600
C19—H19B	0.9600	C39—H39B	0.9600
C19—H19C	0.9600	C39—H39C	0.9600
			
C20—O2—H2B	109.5	C40—O3—H3D	109.5
C2—C1—H1A	120.0	C22—C21—H21A	120.0
C2—C1—H1B	120.0	C22—C21—H21B	120.0
H1A—C1—H1B	120.0	H21A—C21—H21B	120.0
C1—C2—C4	140.4 (11)	C21—C22—C24	128.7 (9)
C1—C2—H2A	109.8	C21—C22—H22A	115.6
C4—C2—H2A	109.8	C24—C22—H22A	115.6
C4—C3—H3A	109.5	C24—C23—H23A	109.5
C4—C3—H3B	109.5	C24—C23—H23B	109.5
H3A—C3—H3B	109.5	H23A—C23—H23B	109.5
C4—C3—H3C	109.5	C24—C23—H23C	109.5
H3A—C3—H3C	109.5	H23A—C23—H23C	109.5
H3B—C3—H3C	109.5	H23B—C23—H23C	109.5
C2—C4—C3	113.4 (7)	C22—C24—C25	108.8 (7)
C2—C4—C5	104.3 (7)	C22—C24—C23	115.0 (7)
C3—C4—C5	113.7 (6)	C25—C24—C23	109.9 (6)
C2—C4—C9	108.4 (6)	C22—C24—C29	108.7 (6)
C3—C4—C9	109.2 (6)	C25—C24—C29	106.1 (6)
C5—C4—C9	107.5 (6)	C23—C24—C29	108.0 (6)
C4—C5—C6	111.0 (6)	C26—C25—C24	115.6 (6)
C4—C5—H5A	109.4	C26—C25—H25A	108.4
C6—C5—H5A	109.4	C24—C25—H25A	108.4
C4—C5—H5B	109.4	C26—C25—H25B	108.4
C6—C5—H5B	109.4	C24—C25—H25B	108.4
H5A—C5—H5B	108.0	H25A—C25—H25B	107.5
C7—C6—C5	111.5 (5)	C25—C26—C27	113.8 (6)
C7—C6—H6A	109.3	C25—C26—H26A	108.8
C5—C6—H6A	109.3	C27—C26—H26A	108.8
C7—C6—H6B	109.3	C25—C26—H26B	108.8
C5—C6—H6B	109.3	C27—C26—H26B	108.8
H6A—C6—H6B	108.0	H26A—C26—H26B	107.7
C8—C7—C6	111.4 (5)	C28—C27—C26	111.7 (6)
C8—C7—C10	113.1 (5)	C28—C27—C30	113.6 (5)
C6—C7—C10	112.3 (5)	C26—C27—C30	114.4 (5)
C8—C7—H7A	106.5	C28—C27—H27A	105.4
C6—C7—H7A	106.5	C26—C27—H27A	105.4
C10—C7—H7A	106.5	C30—C27—H27A	105.4
C13—C8—C7	124.8 (6)	C33—C28—C29	121.3 (6)
C13—C8—C9	120.2 (7)	C33—C28—C27	121.0 (7)
C7—C8—C9	114.8 (6)	C29—C28—C27	117.6 (6)
C8—C9—C4	114.2 (5)	C28—C29—C24	116.2 (6)
C8—C9—H9A	108.7	C28—C29—H29A	108.2
C4—C9—H9A	108.7	C24—C29—H29A	108.2
C8—C9—H9B	108.7	C28—C29—H29B	108.2
C4—C9—H9B	108.7	C24—C29—H29B	108.2
H9A—C9—H9B	107.6	H29A—C29—H29B	107.4
C14—C10—C18	111.2 (6)	C34—C30—C31	111.6 (6)
C14—C10—C11	110.0 (5)	C34—C30—C38	111.1 (6)
C18—C10—C11	112.4 (5)	C31—C30—C38	112.0 (5)
C14—C10—C7	108.0 (5)	C34—C30—C27	107.5 (5)
C18—C10—C7	109.3 (5)	C31—C30—C27	106.5 (5)
C11—C10—C7	105.6 (5)	C38—C30—C27	107.9 (5)
C12—C11—C10	111.5 (5)	C30—C31—C32	111.6 (6)
C12—C11—C17	111.4 (5)	C30—C31—C37	116.7 (5)
C10—C11—C17	117.4 (5)	C32—C31—C37	112.7 (5)
C12—C11—H11A	105.1	C30—C31—H31A	104.8
C10—C11—H11A	105.1	C32—C31—H31A	104.8
C17—C11—H11A	105.1	C37—C31—H31A	104.8
C11—C12—C13	110.2 (5)	C33—C32—C31	110.4 (6)
C11—C12—H12A	109.6	C33—C32—H32A	109.6
C13—C12—H12A	109.6	C31—C32—H32A	109.6
C11—C12—H12B	109.6	C33—C32—H32B	109.6
C13—C12—H12B	109.6	C31—C32—H32B	109.6
H12A—C12—H12B	108.1	H32A—C32—H32B	108.1
C8—C13—C12	122.3 (6)	C28—C33—C32	125.2 (7)
C8—C13—H13A	118.8	C28—C33—H33A	117.4
C12—C13—H13A	118.8	C32—C33—H33A	117.4
C10—C14—C15	112.3 (6)	C30—C34—C35	111.4 (6)
C10—C14—H14A	109.2	C30—C34—H34A	109.3
C15—C14—H14A	109.2	C35—C34—H34A	109.3
C10—C14—H14B	109.2	C30—C34—H34B	109.3
C15—C14—H14B	109.2	C35—C34—H34B	109.3
H14A—C14—H14B	107.9	H34A—C34—H34B	108.0
C14—C15—C16	111.4 (6)	C36—C35—C34	107.8 (6)
C14—C15—H15A	109.3	C36—C35—H35A	110.1
C16—C15—H15A	109.3	C34—C35—H35A	110.1
C14—C15—H15B	109.3	C36—C35—H35B	110.1
C16—C15—H15B	109.3	C34—C35—H35B	110.1
H15A—C15—H15B	108.0	H35A—C35—H35B	108.5
C15—C16—C17	112.0 (6)	C35—C36—C37	114.6 (6)
C15—C16—H16A	109.2	C35—C36—H36A	108.6
C17—C16—H16A	109.2	C37—C36—H36A	108.6
C15—C16—H16B	109.2	C35—C36—H36B	108.6
C17—C16—H16B	109.2	C37—C36—H36B	108.6
H16A—C16—H16B	107.9	H36A—C36—H36B	107.6
C20—C17—C19	112.0 (6)	C40—C37—C39	106.6 (5)
C20—C17—C11	109.0 (5)	C40—C37—C36	105.3 (6)
C19—C17—C11	113.8 (6)	C39—C37—C36	111.6 (6)
C20—C17—C16	104.3 (5)	C40—C37—C31	111.0 (5)
C19—C17—C16	108.4 (6)	C39—C37—C31	113.4 (6)
C11—C17—C16	108.8 (6)	C36—C37—C31	108.6 (5)
C10—C18—H18A	109.5	C30—C38—H38A	109.5
C10—C18—H18B	109.5	C30—C38—H38B	109.5
H18A—C18—H18B	109.5	H38A—C38—H38B	109.5
C10—C18—H18C	109.5	C30—C38—H38C	109.5
H18A—C18—H18C	109.5	H38A—C38—H38C	109.5
H18B—C18—H18C	109.5	H38B—C38—H38C	109.5
C17—C19—H19A	109.5	C37—C39—H39A	109.5
C17—C19—H19B	109.5	C37—C39—H39B	109.5
H19A—C19—H19B	109.5	H39A—C39—H39B	109.5
C17—C19—H19C	109.5	C37—C39—H39C	109.5
H19A—C19—H19C	109.5	H39A—C39—H39C	109.5
H19B—C19—H19C	109.5	H39B—C39—H39C	109.5
O1—C20—O2	121.3 (7)	O4—C40—O3	121.1 (7)
O1—C20—C17	122.6 (6)	O4—C40—C37	123.4 (7)
O2—C20—C17	116.1 (7)	O3—C40—C37	115.4 (6)
			
C1—C2—C4—C3	24.1 (17)	C21—C22—C24—C25	107.4 (11)
C1—C2—C4—C5	148.3 (13)	C21—C22—C24—C23	−16.4 (14)
C1—C2—C4—C9	−97.4 (14)	C21—C22—C24—C29	−137.6 (10)
C2—C4—C5—C6	174.2 (6)	C22—C24—C25—C26	172.6 (6)
C3—C4—C5—C6	−61.8 (7)	C23—C24—C25—C26	−60.7 (9)
C9—C4—C5—C6	59.2 (8)	C29—C24—C25—C26	55.8 (8)
C4—C5—C6—C7	−60.6 (8)	C24—C25—C26—C27	−55.4 (9)
C5—C6—C7—C8	52.2 (8)	C25—C26—C27—C28	42.9 (8)
C5—C6—C7—C10	−179.8 (6)	C25—C26—C27—C30	173.7 (6)
C6—C7—C8—C13	137.7 (7)	C26—C27—C28—C33	144.3 (7)
C10—C7—C8—C13	10.1 (9)	C30—C27—C28—C33	13.1 (9)
C6—C7—C8—C9	−46.6 (7)	C26—C27—C28—C29	−38.6 (8)
C10—C7—C8—C9	−174.3 (5)	C30—C27—C28—C29	−169.8 (5)
C13—C8—C9—C4	−135.3 (7)	C33—C28—C29—C24	−138.2 (7)
C7—C8—C9—C4	48.8 (8)	C27—C28—C29—C24	44.7 (9)
C2—C4—C9—C8	−165.8 (6)	C22—C24—C29—C28	−166.6 (7)
C3—C4—C9—C8	70.2 (8)	C25—C24—C29—C28	−49.7 (8)
C5—C4—C9—C8	−53.6 (8)	C23—C24—C29—C28	68.1 (8)
C8—C7—C10—C14	−159.8 (5)	C28—C27—C30—C34	−165.1 (5)
C6—C7—C10—C14	73.1 (6)	C26—C27—C30—C34	65.1 (7)
C8—C7—C10—C18	79.1 (6)	C28—C27—C30—C31	−45.4 (7)
C6—C7—C10—C18	−48.0 (7)	C26—C27—C30—C31	−175.2 (6)
C8—C7—C10—C11	−42.0 (7)	C28—C27—C30—C38	75.0 (6)
C6—C7—C10—C11	−169.2 (5)	C26—C27—C30—C38	−54.8 (7)
C14—C10—C11—C12	−178.6 (5)	C34—C30—C31—C32	−178.2 (5)
C18—C10—C11—C12	−54.0 (7)	C38—C30—C31—C32	−52.9 (7)
C7—C10—C11—C12	65.1 (7)	C27—C30—C31—C32	64.8 (7)
C14—C10—C11—C17	−48.3 (8)	C34—C30—C31—C37	−46.6 (8)
C18—C10—C11—C17	76.3 (7)	C38—C30—C31—C37	78.7 (7)
C7—C10—C11—C17	−164.6 (5)	C27—C30—C31—C37	−163.6 (5)
C10—C11—C12—C13	−52.7 (7)	C30—C31—C32—C33	−49.6 (7)
C17—C11—C12—C13	173.9 (5)	C37—C31—C32—C33	176.9 (5)
C7—C8—C13—C12	4.0 (10)	C29—C28—C33—C32	−173.5 (7)
C9—C8—C13—C12	−171.4 (6)	C27—C28—C33—C32	3.5 (11)
C11—C12—C13—C8	17.2 (9)	C31—C32—C33—C28	14.2 (10)
C18—C10—C14—C15	−72.3 (7)	C31—C30—C34—C35	55.7 (8)
C11—C10—C14—C15	53.0 (8)	C38—C30—C34—C35	−70.2 (7)
C7—C10—C14—C15	167.9 (5)	C27—C30—C34—C35	172.0 (5)
C10—C14—C15—C16	−60.5 (8)	C30—C34—C35—C36	−62.2 (8)
C14—C15—C16—C17	58.8 (9)	C34—C35—C36—C37	61.0 (8)
C12—C11—C17—C20	−70.0 (7)	C35—C36—C37—C40	−169.2 (6)
C10—C11—C17—C20	159.7 (6)	C35—C36—C37—C39	75.5 (8)
C12—C11—C17—C19	55.8 (7)	C35—C36—C37—C31	−50.3 (7)
C10—C11—C17—C19	−74.5 (8)	C30—C31—C37—C40	157.9 (6)
C12—C11—C17—C16	176.8 (5)	C32—C31—C37—C40	−71.0 (7)
C10—C11—C17—C16	46.5 (8)	C30—C31—C37—C39	−82.1 (7)
C15—C16—C17—C20	−166.5 (6)	C32—C31—C37—C39	49.0 (8)
C15—C16—C17—C19	74.0 (7)	C30—C31—C37—C36	42.5 (7)
C15—C16—C17—C11	−50.2 (8)	C32—C31—C37—C36	173.6 (5)
C19—C17—C20—O1	−152.8 (7)	C39—C37—C40—O4	−121.8 (7)
C11—C17—C20—O1	−26.0 (10)	C36—C37—C40—O4	119.5 (7)
C16—C17—C20—O1	90.1 (8)	C31—C37—C40—O4	2.2 (9)
C19—C17—C20—O2	29.6 (9)	C39—C37—C40—O3	59.8 (8)
C11—C17—C20—O2	156.4 (6)	C36—C37—C40—O3	−58.9 (7)
C16—C17—C20—O2	−87.4 (7)	C31—C37—C40—O3	−176.3 (6)

### Hydrogen-bond geometry (Å, °)

**Table:**

*D*—H···*A*	*D*—H	H···*A*	*D*···*A*	*D*—H···*A*
O2—H2B···O4^i^	0.82	1.84	2.653 (7)	168
O3—H3D···O1^ii^	0.82	1.85	2.655 (7)	168
C11—H11A···O1	0.98	2.34	2.764 (7)	105
C32—H32B···O4	0.97	2.49	3.081 (9)	119

Symmetry codes: (i) *x*+1/2, −*y*+5/2, −*z*+2; (ii) *x*−1/2, −*y*+5/2, −*z*+2.
